# Evolution of Antimicrobial Peptides to Self-Assembled Peptides for Biomaterial Applications

**DOI:** 10.3390/pathogens3040791

**Published:** 2014-10-03

**Authors:** Alice P. McCloskey, Brendan F. Gilmore, Garry Laverty

**Affiliations:** Biomaterials, Biofilm and Infection Control Research Group, School of Pharmacy, Queen’s University Belfast, Medical Biology Centre, 97 Lisburn Road, Belfast BT9 7BL, N. Ireland; E-Mails: amccloskey16@qub.ac.uk (A.P.M.); b.gilmore@qub.ac.uk (B.F.G.)

**Keywords:** antimicrobial, bacteria, biofilm, biomaterial, infection, peptide, self-assembly

## Abstract

Biomaterial-related infections are a persistent burden on patient health, recovery, mortality and healthcare budgets. Self-assembled antimicrobial peptides have evolved from the area of antimicrobial peptides. Peptides serve as important weapons in nature, and increasingly medicine, for combating microbial infection and biofilms. Self-assembled peptides harness a “bottom-up” approach, whereby the primary peptide sequence may be modified with natural and unnatural amino acids to produce an inherently antimicrobial hydrogel. Gelation may be tailored to occur in the presence of physiological and infective indicators (e.g. pH, enzymes) and therefore allow local, targeted antimicrobial therapy at the site of infection. Peptides demonstrate inherent biocompatibility, antimicrobial activity, biodegradability and numerous functional groups. They are therefore prime candidates for the production of polymeric molecules that have the potential to be conjugated to biomaterials with precision. Non-native chemistries and functional groups are easily incorporated into the peptide backbone allowing peptide hydrogels to be tailored to specific functional requirements. This article reviews an area of increasing interest, namely self-assembled peptides and their potential therapeutic applications as innovative hydrogels and biomaterials in the prevention of biofilm-related infection.

## 1. Introduction

Biomaterials have an increasingly important role in patient care. Approximately five million medical devices and implants are used in the US each year [1]. They are beneficial not only as temporary interventions but also as permanent features to replace or facilitate normal bodily functions. There is a diverse array of such materials available including: intravascular and urinary catheters, heart valve prostheses, artificial hip joints, dental implants, and intraocular lenses [2]. Their use is particularly prominent in modern medicine as population demographics demonstrate an increasing trend toward an ageing population, in tandem with increased life expectancy and improved healthcare [3].

Grainger and colleagues researched the contribution medical devices have to improving patients’ quality of life [4]. They concluded that 50% of patients with aortic heart valve disease could potentially die within three years of diagnosis. Valve replacements resulted in an increase in ten year survival to approximately 70% of patients. Despite this improvement, 60% of these patients were shown to have device-associated complications thus affecting patient quality of life. Implantation of a medical device is an invasive surgical procedure that may result in irritation, wounding and infection of the surgical site. A macrophage-mediated collection of immune responses, collectively referred to as the host or foreign body response, is triggered leading to compromised device function and scarring of surrounding tissue [5]. Anderson and colleagues provide a comprehensive outline of the sequence of events following surgical implantation [6]. The initial response follows the normal wound-healing cascade of: injury, blood-material interactions, provisional matrix formation, acute inflammation, chronic inflammation, granulation tissue development, foreign body reaction, and fibrosis. The resulting host or foreign body response to a device is estimated to affect approximately 5% of patients and can have a significant impact on patient recovery and long-term health. Surgical trauma can result in a compromised immune response leading to unchallenged accumulation and adherence of pathogens at the site of implantation, resulting in biofilm formation. Biomaterials provide an optimal surface for biofilm formation [1,7]. The long-term effects are considerable. Economically, biomaterial associated infections have costly implications on health-care budgets. Bacterial accumulation and the subsequent formation of a biofilm contributes to an infectious profile 10–1000 times more resistant to standard therapeutic regimens, leading to compromised implant function and/or failure [8]. Symptomatic infection often occurs within 2 weeks of implantation [4,6,7,9].

Biofilms are phenotypically heterogeneous, sessile microbial communities that form at biotic and abiotic sites. They may be composed of a single or multiple species (poly-microbial) of bacteria and fungi [10,11,12]. Biofilm formation can be divided into three significant stages: (1) initial non-specific, reversible primary adhesion; (2) specific irreversible adhesion (coating of the device, transport of cells to the interface, adhesion of cells; accumulation at the surface involving biomolecular processes including quorum sensing, up-regulation of virulence factors and secretion of extracellular polymers; (3) biofilm detachment/dispersal and recolonization of alternative sites. Following implantation the surface chemistry of a biomaterial is modified by the laying down of a host-derived conditioning film. Primary adhesion is dependent on two key factors—hydrophilicity and charge at the cell-device interface. Primary adhesion is a non-specific process mediated by hydrophobic and van der Waal’s interactions between the microbial cell and the generally hydrophobic surface, as is the case for silicone [1,10,11]. Binding sites such as fibrinogen, fibronectin, vitronectin, binding autolysin, albumin and extracellular matrix binding protein become exposed on the biomaterial surface. Microbial adhesins, for example teichoic acid expressed in *Staphylococcus epidermidis*, specifically recognize binding sites and mediate adherence to the surface via covalent attachment.

The potential for microbial spoilage is not confined to hydrophobic surfaces. More hydrophilic materials, for example Teflon^®^, also suffer from microbial contamination due to mechanical rather than chemical factors [13]. MacKintosh and co-workers investigated how the modification of poly(ethylene terephthalate) surface chemistry affected bacterial-biomaterial interaction, attachment and subsequent biofilm formation of *Staphylococcus epidermidis* [14]. They demonstrated that hydrophobic interactions do influence the adhesion of bacteria to the material surface. Of greater significance is the presence of serum proteins within the conditioning film. These tend to have a greater effect on adhesion and biofilm formation *in vivo*. Similar research performed by the Gottenbos group suggested that biofilm formation is a complex relationship involving not only the biomaterial surface properties and the ability of the bacteria to bind to a given surface, but also the availability of nutrients at the site of adherence [15].

Accumulation on a surface occurs when bacterial cells multiply and form multi-layered cell clusters resulting in a complex three dimensional architecture- the classic biofilm [16]. The biofilm is maintained through specific quorum sensing pathways. Quorum sensing is a communicatory and regulatory process controlling the up- and down-regulation of genes that govern virulence and adhesion factors, relative to environmental conditions and population density. It is an essential process for biofilm survival ensuring that biofilms act as a mutualistic community rather than individual cells [17]. As the microbial population increases the supply of nutrients and oxygen become limited within the confines of the biofilm. Therefore biofilm bacteria generally grow more slowly than planktonic bacteria due to a reduced rate of respiration/metabolism. The protective environment, provided by the polymeric exopolysaccharide matrix, means that bacteria are much more resistant to the host’s immune response and therapeutic antimicrobials than their planktonic counterparts [1,18,19]. Biofilm mediated infections are thus extremely difficult to treat and the extent and rate of eradication is largely determined by the most resistant phenotype within the biofilm. Chemotherapeutic failure often results in surgical removal of the biomaterial, particularly those involving *Staphylococcus aureus* and *Candida* species [7]. Removal, combined with potential chemotherapeutic failure, is not an ideal scenario as concerns grow regarding increased antimicrobial resistance, and the relative lack of new antimicrobials in development [20,21,22].

Typical biofilm related medical device infections include: Catheter associated urinary tract infections; peristomal skin infections following insertion of percutaneous endoscopic gastrostomy feeding tubes; and pneumonia or tracheobronchitis with tracheostomy devices. The most commonly implicated pathogens are: staphylococci, enterococci, *Escherichia coli*, *Proteus mirabilis*, *Pseudomonas*, and *Candida* [19,23,24]. The prognosis for such infections depend on the patient’s initial health, which in many cases is poor due to age and co-morbidities, and the duration of implantation. Medical implants are commonly required in immunocompromised patients. Therefore, insertion of an implant and the resulting trauma further compromises the immune response increasing patient recovery time and morbidity.

Numerous strategies to reduce biomaterial-associated infections have been developed but few have translated to clinical practice [9]. Hospital stays can be up to two and a half times longer than for uninfected patients, with a total of 3.6 million extra days being spent in hospital per year in England. Nosocomial infections cost the health sector in England almost £1 billion per year [25]. In the United States medical device related infections contribute to over 50,000 deaths per year [4]. As for the majority of disease states prevention is the key aim. Poor hygiene practice within the healthcare setting has been shown to increase the risk of infection. Simple measures such as correct hand washing technique, by both staff and patients, can have a dramatic decrease in infections [26]. There is an increasing demand globally for medical devices to replace normal physiological function. Therefore managing and preventing implant associated infections is a huge challenge [1]. These problems have to be addressed on a global-scale and require the development of biomaterials that are both biocompatible and anti-infective. This review examines the current strategies employed to reduce the occurrence of biofilm mediated device related infections and investigates the potential of future innovative strategies, namely peptide based biomaterials.

## 2. Current Research Based Strategies for the Prevention of Medical Device Related Infection

Biomaterials cover a diverse range of pharmaceutical applications from drug delivery to tissue engineering [27]. Every device is prone to infection. Surfaces are particularly vulnerable to biofilm formation. Therefore antimicrobial coatings are a plausible solution for the development of devices with anti-infective properties. Implantation of medical devices may be classified as temporary or permanent/long-term. Temporary devices, for example contact lenses, are not fully integrated into the host tissue. Other internally-based devices, for example heart valves, tend to be more permanent. Prevention of temporary device-related infections can be managed with non-adhesive, antimicrobial impregnated or releasing coatings, which kill bacteria that come into contact with the device [9]. Permanent device coatings must be multi-functioning, facilitating incorporation of the device into the host tissue whilst simultaneously preventing microbial adhesion over an extended period within the lifetime of the device. Examples of such coatings include those investigated by the Saldarriaga group [28]. They produced multi-component cross-linked poly(ethylene-glycol) based polymers and demonstrated that the degree of hydration and steric hindrance contributed to the efficacy of these multi-functioning coatings. Hydrogel coatings display great promise as they can incorporate and/or release antimicrobial agents. They allow for improved tissue integration and a reduction in biofilm formation [29]. Hydrogels comprise a group of insoluble, swellable, hydrophilic polymers. When fully swollen they are composed of a significant amount of water (up to ~99%) but also display solid-like properties which provide desirable characteristics such as increased mechanical strength. Hydrogel classification is dependent upon the nature of the crosslinks which bind the hydrogel structure, influencing its swelling ability. Chemical hydrogels are also influenced by the structure of the primary monomer chains and the density of the crosslinks within the polymer system. Hydrogel architecture is also determined by secondary non-covalent molecular interactions and entangled molecules. The presence of a porous three-dimensional network means hydrogels are ideal biomaterials as they structurally similar to the extracellular matrix and tissue. The presence of defined functional groups, for example carboxylic acids, allow for the production of bioactive biomaterials that respond to environmental, chemical and physical stimuli. These so-called ‘smart’ polymer systems display significant potential as future drug delivery and biomaterial platforms [30,31,32].

Current research has seen an increased focus on the antimicrobial properties of silver. Hydrogel/silver coated urinary catheters have been investigated and promising results obtained. A reduction in the primary bacterial adherence was observed in comparison to a standard silicone catheter indicating that hydrogel/silver coatings have the potential to delay the onset of catheter associated infections [33]. Synergistic combinations of antibiotic(s) and/or antiseptics are also utilized clinically to reduce the incidence of infection. ARROWgard™ central venous catheters are coated by a combination of silver sulphadiazine and chlorhexidine, whilst the commercially available alternatives BioGuard Spectrum™ and Cook Spectrum^®^ are both coated by a combination of minocycline and rifampicin [34]. Silver sulphadiazine and chlorhexidine coated catheters displayed significant activity against a range of microorganisms including *Candida albicans* and *Escherichia coli* for up to seven days [35]. Minocycline-rifampicin catheters were shown to display only bacteriostatic activity against slime producing forms of *Staphylococcus epidermidis* (ATCC 35984) and *Staphylococcus aureus* (ATCC 29213) but for an extended period of up to 21 days [36]. A range of other anti-infective biomaterials are available commercially, aiming to deliver antimicrobials locally at the device surface for the prevention of biofilm formation. Many challenges exist with this strategy. It is difficult to ensure the dose of antimicrobial delivered is uniform within the vicinity of the device surface. Areas of the implant may be exposed to sub-therapeutic concentrations of antimicrobials [37]. Sub-inhibitory concentrations of antimicrobial agents may lead to increased microbial resistance. Research by Rachid highlighted this effect whereby an increase in *ica* operon expression [38], linked with staphylococcal polysaccharide intercellular adhesin accumulation [39,40], occurred in response to sub-optimal levels of the antibiotic tetracycline and the semi-synthetic molecule quinuprisin- dalfopristin.

One of the major limitations regarding the delivery of antimicrobials in a biomaterial model is the effect of burst-release. Burst-release is consistent with an initial high and rapid release of the antimicrobial from the biomaterial. It is one of the major challenges of modern drug delivery. The antimicrobial reservoir depletes to sub-inhibitory levels within days allowing infection to develop unchallenged. Covalent attachment of antimicrobial, as outlined by the examples of ARROWgard™, BioGuard Spectrum™ and Cook Spectrum^®^, suffer from a reduction in activity due to masking of antimicrobials by the host’s conditioning film [41]. A significant profile of prolonged release over weeks or an infection responsive system is more desirable [42]. A multitude of studies exist in antimicrobial drug delivery to resolve this issue. Examples include sustained release systems such as calcium-mediated delivery of the broad-spectrum antibiotic minocycline, as demonstrated by Zhang and colleagues [29]. The authors utilized layer-by-layer assembly and calcium binding to create a sustained release platform. Delivery of minocycline is due to a local change in pH in the vicinity of the device. Acidosis present in the tissues as a result of medical device induced inflammation or infection, weakens the chelation reaction between minocycline and calcium ions, resulting in subsequent release of minocycline. This so-called ‘smart’ approach to drug delivery shows promise for future biomedical application.

There has also been increasing interest in using peptides for biomaterial applications [43]. As antimicrobials, peptides serve as barriers to infection throughout nature as part of the innate immune response [44]. There are a multitude of examples whereby antimicrobial peptides have been synthesized to disrupt microbial biofilms [45,46,47]. Peptides also display a diverse array of properties making them suitable for biomaterial applications including: increased biocompatibility and minimal immunogenicity; the availability of moieties for functionalization; chemical versatility and biodegradability [48]. Peptides have also demonstrated an ability to self-assemble into supramolecular hydrogels in response to environmental stimuli for example pH, the presence of salts, heat and enzymatic cleavage [49,50]. These characteristics may allow such compounds to be utilized to form inherently antimicrobial peptide hydrogel structures in response to infection.

## 3. Current Approaches to Self-Assembling Biomaterials

The process of self-assembly is an important parameter for the development of novel biomaterials. It is particularly relevant with regards to nanotechnology. These principles have been adopted from nature. Ribosomes and the quaternary haemoglobin structures are examples of naturally occurring self-assembled architectures [51]. Assembly involves the spontaneous arrangement of pre-existing, disordered molecules of similar properties, to form higher ordered structures. It is mediated by non-covalent, local interactions: van der Waals forces, hydrogen bonding, π-π stacking, and electrostatic interactions [52,53]. Movement at a molecular level facilitates assembly. Environmental factors such as volume and binding influence assembly. Equilibrium between the aggregate and non-aggregate states is essential to maintain a higher ordered structure [54,55]. Self-assembly has recently attracted heavy investment from both the private and public sectors. In 2010 global public investment was estimated at approximately $8.2 billion and private sector funding slightly higher at $9.6 billion [56].

Current polymer technologies include the area of Pluronics or poloxamers. Composed of three polymers polyoxyethylene (PEO), polyoxypropylene (PPO) and polyoxyethylene (PEO), Pluronics is an area that has been extensively studied with regard to micellization and the formation of structures for drug delivery purposes. Pluronics have the ability to assemble at physiological temperatures, in a variety of solvents, and over a range of concentrations as opposed to a single critical concentration [57]. There is a great deal of interest within the drug delivery field in exploiting gels that have the ability to flow at ambient temperatures but form a gel upon exposure to physiological temperatures. These gels have great potential in terms of targeted or localized drug delivery of anticancer and antimicrobial drugs in particular [58]. Self-assembling Pluronics are examples of non-ionic and non-toxic gels [59]. F127 is an example of a Pluronic polymer whereby assembly is triggered due to temperature change. Its ability to form micelles at human body temperature means that F127 has potential in the delivery of poorly soluble, hydrophobic drugs [57]. Sustained release of drugs from Pluronics has been widely investigated. Barichello’s group used F127 alone and in addition to poly-co-glycolic acid (PLGA) nanoparticles for protein delivery, using insulin as a model drug [60]. They demonstrated F127 incorporated insulin has the potential to provide a controlled release system. The short-acting, opioid analgesic fentanyl has also been delivered using gels formed by PEO-PPO-PEO. Investigations have shown to demonstrate a similar release flux to the commercially available Durogesic^®^ patch [59]. The possibility exists that this type of delivery could be adopted for sustained delivery of antimicrobials to wounds or burns, removing the need for frequent changes of dressings. The antibiofilm activity of Pluronics has been studied due to its non-ionic surfactant-like properties [61]. Wesenberg-Ward and colleagues discovered that Pluronic F127 conditioned polystyrene reduced *Candida albicans* biofilm formation relative to untreated polystyrene controls [62]. Reduction in *Staphylococcus aureus* and *Staphylococcus epidermidis* adherence to polymethylmethacrylate and increased susceptibility to vancomycin and gentamicin was also observed in the presence of poloxamer 407 [63]. These studies highlight the potential use of Pluronics in the biomaterials field to produce surfaces that are resistant to microbial adhesion. Recent research by Leszczyńska outlined the use of Pluronic F127 topical antimicrobial in combination with the synthetic cationic antibacterial peptide Ceragenin CSA-13 [64]. In this instance, Pluronic F127 acted as the drug delivery vehicle with broad spectrum activity demonstrated against methicillin-resistant *Staphylococcus aureus* and a *Pseudomonas aeruginosa* strain isolated from cystic fibrosis patients. Pluronics have also been linked with increased wound-healing in animal models validating continuous research into their applications as effective biomaterials [65].

Related research combines innovative approaches to stimulate antimicrobial release with self-assembled hydrogel polymers. Norris and colleagues synthesized a self-assembled poly(2-hydroxyethyl methacrylate) coating containing ordered methylene chains [66]. This polymer released ciprofloxacin in response to low intensity ultrasound and displayed significantly reduced accumulation of established 24 h *Pseudomonas aeruginosa* biofilm over a three day period. Self-assembled monolayers have been created to modify the surface properties of biomaterials with the aim of reducing bacterial attachment. Recently Kruszewski *et al*. produced stainless steel, commonly employed in orthopaedic implants, modified by a self-assembled monolayer of long alkyl chains terminated with hydrophobic (−CH_3_) or hydrophilic (oligoethylene glycol) tail groups [67]. These groups facilitated the attachment of gentamicin or vancomycin and reduced *Staphylococcus aureus* biofilm formation for up to 24 and 48 h respectively. Protein deposition by the host conditioning film remains a problem due to masking of covalently attached molecules. Studies exist for self-assembled monolayer materials that display resistance to biofilm formation and host protein adsorption. Self-assembled monolayers of alkanethiols, presenting a tri(ethylene glycol) functional group, displayed a profile of reduced protein adsorption and mammalian cell adhesion and resisted *Escherichia coli* biofilm formation [68]. The authors concluded this may be due to the ability of tri(ethylene glycol) to repel cells and inhibit bacterial cell motility, a key factor in biofilm formation. Silicon coated with a self-assembled micro-gel consisting of poly(ethylene glycol) and poly(ethylene glycol)-*co*-acrylic acid proved resistant to adhesion by *Staphylococcus epidermidis* for up to 10 h [69]. Loading the micro-gel with an antimicrobial peptide (L5) resulted in significant anti-adherent properties at the 10 h time-point due to the localized release and inhibitory action of the L5 peptide. After 10 h, colonization was observed due to depletion of the peptide reservoir.

Such approaches have been significant in advancing biofilm-resistant self-assembled polymer research. Limitations exist affecting translation into clinical practice, namely sufficient antimicrobial action over a sustained period of time. Self-assembled polymers have contributed to a multitude of innovative investigations and applications within biomedical engineering. It is now a widely accepted method of material development. The theory has been expanded to investigate the potential of peptide self-assembly and peptide-based nanomaterials. Self-assembled peptides display great promise as antimicrobial coatings. Alteration of the peptide backbone allows materials to be tailored to their application. The production of an inherently antimicrobial peptide hydrogel, which self-assembles in response to environmental or specific pathogenic stimuli, may allow for significant reduction of biofilm formation over a prolonged period.

Uses for self-assembled peptides range from medical to engineering applications. They have recently come to the fore of bioscience contributing significantly to the nanotech revolution [70]. Work by Zhang in 1993 on ionic self-complementary peptides is regarded as particularly noteworthy in terms of evolving this area [71]. Other experts of note include: Aggeli, who investigated the hierarchical structures of peptides [72]; Tirrell, who patented secondary structure forming peptide amphiphiles [27,73]; and Ghadiri, who was responsible for the development of cyclic nanopeptides [74]. To fully understand the potential of self-assembling peptides in biomaterial applications an appreciation of the chemical composition governing their hierarchical properties is required.

**Figure 1:**
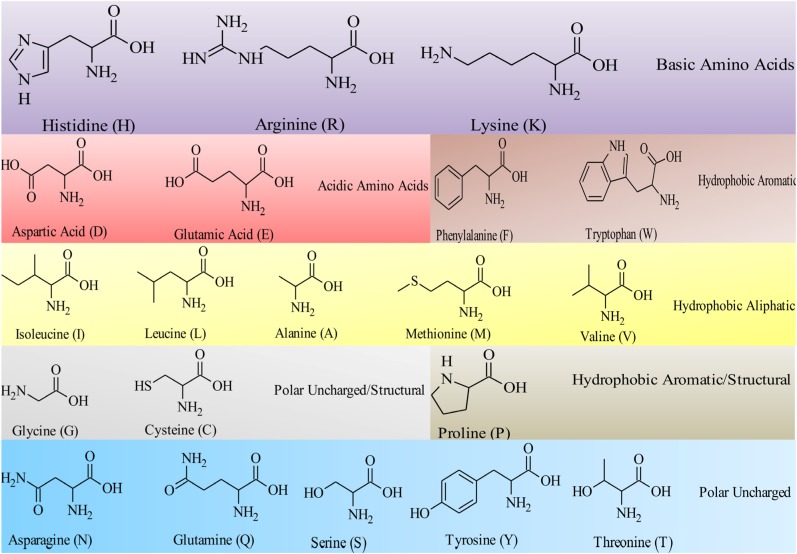
The structures and single amino acid code for the twenty naturally occurring amino acids. Each amino acid shares a carboxylic acid (−COOH) and a primary amine group (−NH_2_). The properties of the individual amino acids are governed by the nature and functionality of the R-group attached to the α-carbon. Researchers exploit the differences in individual amino acid units to develop peptide-based therapeutics. Of particular importance to antimicrobial peptides and peptide self-assembly is the hydrophobic: hydrophilic balance of the primary peptide structure.

Peptides are ideal building blocks for the formation of nanostructures. Utilizing a “bottom up” approach, individual amino acid residues are used to build higher ordered structures [75]. The twenty naturally occurring amino acids alone allow a huge variety of potential combinations. Their structures and corresponding single letter abbreviation are detailed in Figure 1. An immense range of primary peptide sequences and higher ordered structures can be created for biomedical applications. Variation of the R-group at the alpha (α) carbon provides unique characteristics (hydrophobic, hydrophilic, aliphatic, aromatic, positive or negative charge) to each amino acid. These influence the extent to which the molecules can participate in non-covalent interactions and therefore self-assemble to form defined nanostructures. The hydrophobic: hydrophilic balance and ability of these molecules to hydrogen bond are particularly influential factors [55]. Ulijn and Smith provide detail regarding the effect of various amino acids on the process of assembly [76]. Their review refers to the relationship between an increase in methylene chains on the R-group side chain; the degree of hydrophobicity; and extent of steric hindrance in affecting assembly of monomer units. In particular, the degree of hydrophobicity is key in determining assembly. Peptide assembly is also influenced by the concentration of peptide present. Increasing the number of amino acid repeats results in the formation of higher assemblies. The number of amino acids present also affects the critical concentration of assembly. The critical concentration increases relative to a rise in the number of amino acid residues due to a shift in the entropic-enthalpic balance. For assembly to occur entropic loss must be balanced with an enthalpic gain within the system [77]. Environmental factors such as pH, temperature, light, and the presence of proteolytic enzymes influence assembly. Peptide systems commonly follow those found in nature. The greater the number of hydrophobic residues present, the lower the critical concentration for assembly to occur. Crucially the degree of charge and hydrophilicity must be optimal to ensure efficient assembly. An increase in hydrophobicity above optimal levels may result in precipitation of the peptide molecule in solution. Hydrophilic groups, including those present within the peptide bond (−NHCO), carboxylic acids (−COOH) and amines (−NH_2_) can also contribute to assembly via hydrogen bond intermolecular interactions with surrounding solvent and neighboring peptide molecules. Charged amino acids, for example lysine, result in charge-charge electrostatic interactions. These have potential to drive or prevent assembly depending on the degree of polarity and charge density. Salt concentration is also a contributing factor, as demonstrated through work on the peptide EAK16-II by Hong and colleagues [78]. A low salt concentration resulted in a desired level of intermolecular interaction, driving assembly. Ionisation, which is strongly linked to pH and p*K*a, has a large influence on the self-assembly process. The natural amino acid tyrosine allows further functionalization of peptide molecules. Its R-group consists of an amphiphilic phenol grouping which allows, for example, formation of imines under relatively mild conditions (pH ~6.5) [79]. The use of microbial enzymes to facilitate self-assembly of antimicrobial peptides is covered in detail in Section 5.4. The phenol grouping of tyrosine provides an alternative functional group for conjugation of possible microbial enzyme targets, for example phosphate groupings [80].

Peptide derivatives as detailed include amphiphiles and π-stacked aromatic peptides. Amphiphiles have a similar structure to membrane phospholipids, possessing a hydrophobic alkyl tail conjugated to a charged moiety. They are ideal molecules for interacting with and disrupting bacterial membranes [81]. Assembly results in a variety of higher structures, mainly rods, which possess a hydrophobic core and a hydrophilic exterior. The process of assembly is modified by altering the charge of the residues involved or by changing the position of the alkyl chain on the alternative termini (amine or carboxylic acid functional groups) [82]. Conjugated amphiphilic peptides with assembling properties for example IKVAV, have been employed in tissue engineering where the assembled nanofibrous network created scaffolds facilitating differentiation of cells into neurons [83].

**Figure 2:**
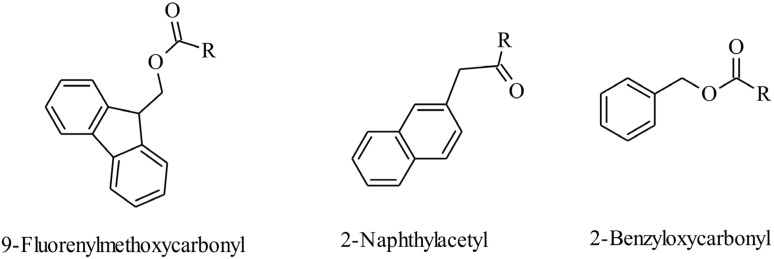
Examples of aromatic moieties utilized to provide π-π electrostatic interactions. The polycyclic aromatic hydrocarbon residues of fluorene (in 9-fluorenylmethoxycarbonyl groups), naphthalene (as in 2-naphthylacetyl) and benzene (as in 2-benzyloxycarbonyl) facilitates π-stacking due to the presence of a conjugated system of delocalized π-electrons. Such intermolecular interactions are enhanced by the presence of hydrophobic aromatic amino acid molecules, for example phenylalanine.

Reviews such as those by Gazit and Martinez [84,85] provide more detailed information regarding π-stacked peptides. In brief, π-stacked peptides are composed of residues with aromatic groups such as phenylalanine, tryptophan, tyrosine and histidine. Burley and Petsko [86] initially investigated the aforementioned, naturally occurring aromatic amino acids, and their interactions with neighboring aromatic moieties. They highlighted the influence of a residue’s environment on the availability of free energy to interact with other residues, and the contribution of this to the resulting structural stability. More recently π-π interactions particularly diphenylalanine interactions, are attracting a great deal of interest. They have a key role in β-amyloid polypeptide/ fibril formation and subsequent formation of fibrous plaques, which are implicated in disease states such as Alzheimer’s [87]. π-π stacking may also be attributed to peptides that have a 9-fluorenylmethoxycarbonyl (Fmoc), naphthalene (Nap), or benzyloxycarbonyl (Cbz) group on the amine terminal (Figure 2). π-stacking occurs due to the presence of a conjugated system, allowing the aromatic π electrons within this system to overlap and interact [88]. The restricted geometries within the aromatic systems provide direction to the assembled structure, with interactions between the stacks acting as a glue and contributing to the overall rigidity of such assembled structures [84].

Although the aim of assembly is to achieve higher-ordered structures, these must be uniform, homogenous and reproducible with defined and useful properties. Peptides provide an ideal basis for such structures for many reasons. They are biocompatible and biodegradable [89]. Their synthesis is relatively uncomplicated [90]. They can assemble (forming gels) *in situ* [71]. Peptide bioactivity, such as antimicrobial potency, can also be modified according to the amino acid residues selected and structure activity relationships can often be determined with high levels of precision. Aromatic groups contribute to π-π intermolecular van der Waal’s interactions. Molecules that contain such groups act as hydrogelators by supplying increased hydrophobic bulk to the molecule. Fmoc, naphthalene, and Cbz may facilitate assembly at a reduced peptide length and cost. Naphthalene is preferred due to an established safety profile as it is used in many licensed drug molecules including propranolol. The cytotoxicity of naphthalene-based peptides was previously investigated by Yang and co-workers [89]. They determined a cell survival of almost 100% when HeLa cells were exposed to 200 micromolar of naphthalene dipeptides. Supramolecular properties of these structures including electrical, mechanical and bioactivity are also determined by the process and kinetics of self-assembly. Therefore the nature of the route and rate of assembly must also be carefully considered when designing self-assembling peptide systems.

## 4. Antimicrobial Peptides

The chemical, physical and structural properties that govern peptide self-assembly are closely associated with factors that determine the antimicrobial potency and spectrum of activity of antimicrobial peptides. Antimicrobial peptides occur throughout nature and are involved in the innate immune response. This response is mounted a short time following exposure to an infective agent and stimulation of cells known as Toll-like receptors [91]. Unlike conventional antimicrobials, most naturally occurring antimicrobial peptides act synergistically in great numbers and at micromolar concentrations. The combined effect of this is a relatively potent antimicrobial response due to action at multiple sites, in comparison to a single target as is the case with most conventional antibiotics [92].

The amphiphilicity of antimicrobial peptides allows them to target microbial cell membranes, particularly those of bacterial cells. Bacterial membranes possess an overall negative charge, compared with neutrally charged eukaryotic membranes, due to the presence of acidic hydroxylated phospholipids. These include cardiolipin, phosphatidylglycerol and phosphatidylserine [93]. The presence of phosphate groups on membrane-bound lipopolysaccharides in Gram-negative bacteria and acidic polymers, such as teichoic acids, in Gram-positive bacteria, allow areas of dense anionic charge to develop on bacterial membranes [44]. Cationic peptides target these areas to exert their effect. Antimicrobial peptides act directly on the cell membrane by disrupting the integrity or function of the phospholipid bilayer via four recognized mechanisms: the aggregate, toroidal pore, barrel-stave and carpet models [94]. Bacterial membranes are an excellent antimicrobial target as bacteria would be required to alter the overall properties of their membrane to confer resistance characteristics, rather than modifying individual receptors. The likelihood for bacteria developing resistance is significantly decreased as antimicrobial peptides also possess intracellular targets. Peptides have been proven to inhibit ATP-dependent enzymes and disrupt processes within the cell, for example RNA and protein synthesis, DNA replication and protein folding [95,96,97,98,99].

Cationic peptides are the most studied form of antimicrobial peptides. Possessing a net positive charge, they are most commonly composed of 12–50 amino acids, approximately 50% of which are hydrophobic [100]. Cationic antimicrobial peptides were first derived from insect haemolymph in the 1970s. They are amphiphilic in nature enabling them to interact with the dense phospholipid exterior of bacterial cell membranes [95]. Cationic peptides have been referred to as ‘nature’s antibiotics,’ however the term “defence proteins” may be more appropriate due to their role in modulating the innate immune response [101]. Anionic peptides are also found throughout nature and have a role in providing innate immunity in a variety of animals and plants. Typically, composed of 7–50 residues, they are also amphiphilic in nature but have an overall net negative charge due to the presence of aspartic acid residues [102,103]. Anionic peptides often require cationic co-factors, for example zinc ions, to produce an antimicrobial effect. Brogden hypothesized zinc ions may form a cationic salt bridge between anionic antimicrobial peptides and the anionic microbial cell surface [104]. These interactions facilitate movement of such peptides across the bacterial membrane, into the cell cytoplasm, allowing intracellular attack. Epithelial cells are prime locations for host defense anionic peptides, not only are they key sites for ion exchange but they are also commonly exposed to pathogenic microbes [105].

Antimicrobial activity and self-assembly of peptides are influenced by similar factors namely: charge, bulk and lipophilicity. Strøm and co-workers investigated the contribution of these factors to antimicrobial activity [106]. Their findings showed that an optimal balance exists between degree of hydrophobicity or bulk and charge governing antimicrobial potency and spectrum of activity. Modification of the peptide sequence with unnatural molecules possessing amino and/or carboxylic acid groups may be of benefit in increasing the cost-effectiveness and activity of the peptide. For example, the addition of cinnamic acid has been shown to increase antimicrobial activity by providing hydrophobic bulk to the primary peptide sequence, at a reduced cost compared with commercially available hydrophobic amino acids [107]. Hydrophobicity and antimicrobial activity can be optimized sequentially via the addition of fatty acids to the peptide motif, creating the promising antimicrobial lipopeptides [45,108]. The type and nature of amino acids employed to generate peptide motifs are also important. Utilizing unnatural amino acids, for example ornithine, may provide the peptide with stability against proteases which are unable to be recognized at a molecular level for incorporation into enzyme active sites [107]. Most amino acids in nature are present as the l-stereoisomer form. Stereoisomerism is based on the ability of molecules to rotate plane-polarized light. l-amino acids are easily recognized by host and bacterial enzymes. They are ideal substrates for proteolysis by peptide specific enzymes termed proteases. In theory this limitation can be reduced, to increase both therapeutic efficacy and retention within the host, by replacement with d-amino acids. Amino acids that are in the d-enantiomeric form do not fit in the active site of proteases and therefore cannot be broken down. d-amino acids do have limitations. They are typically expensive, and for short peptides the realistic enzyme stability they offer is debatable [109].

Lipopeptides are a form of antimicrobial peptides present in nature among bacteria and provide selective advantage against competing microbial strains. Their success is reflected in the pharmaceutical industry, with antimicrobial lipopeptides already successfully utilized clinically. These include anionic daptomycin [110], which forms micelles in the presence of calcium ions facilitating membrane interaction, and cationic polymyxins which interact with membrane-bound lipopolysaccharides inserting into bacterial membranes causing membrane disruption [111]. Cost and toxicity has rendered their use restricted to mainly topical application and second or even third line treatments for pathogens resistant to other antimicrobials. A research paper by Levine presented the findings of patients registered on the Cubicin**^®^** Outcomes Registry and Experience (CORE) 2004 database. This covered patients administered with the injectable form of daptomycin (brand name: Cubicin**^®^**) for the treatment of infective endocarditis [112]. Of those treated, 83% of *Staphylococcus aureus* infections were methicillin resistant, and 43% enterococci infections were vancomycin resistant. The findings showed that daptomycin has a potential role in treating these types of infections, with 63% of treatments proving successful overall. Similarly a review by Li highlights in great detail the effective role of polymyxin E (colistin) via inhalation and intravenous administration to treat multi-resistant pseudomonal infections in cystic fibrosis patients [113]. There is a clinical need to improve toxicity, bioavailability, and selectivity profiles. Activity is influenced by the amino acid chain and hydrophobicity, with the length of the conjugated fatty acid chain an influential factor [91].

Research is increasingly focused on the action of antimicrobial peptides against resistant biofilm phenotypes [114]. Evidence shows that peptides interact with polysaccharide components of the biofilm causing break down within the polymeric matrix [115]. Peptides also act synergistically with standardly employed antimicrobials, eradicating the biofilm and membrane barriers, allowing clinically employed antibiotics access to intracellular targets. The antimicrobial peptides magainin-II (amphibian-derived) and cecropin-A (insect-derived) were proven by Cirioni *et al.* to act synergistically with rifampicin against multi-drug resistant *Pseudomonas aeruginosa* in both *in vivo* and *in vitro* models [116]. The lipopeptide polymyxin E demonstrated synergistic efficacy with ticarcillin/clavulanate against *Stenotrophomonas maltophilia* isolated from cystic fibrosis patients [117]. Infectious diseases and their causative pathogens continue to display a profile of increased resistance to standardly employed antimicrobials. A recent investigation in a Spanish hospital urology department showed that approximately 52% of isolated *Escherichia coli* and 36% *Pseudomonas aeruginosa* displayed resistance to fluoroquinolones [118]. There is a pressing need not only to develop new antimicrobials but also to refine the properties of these potentially novel chemotherapeutics so that stability, size, immunogenicity, and cost-effectiveness can be improved [119]. The area of antimicrobial peptides hold much promise in the development of antimicrobial therapeutics and their potential is covered in more detail in reviews by Bahar [47] and our own research group [44].

Ultrashort refers to peptides up to 7 amino acids in length. They have become increasingly attractive in the development of peptide therapeutics due to reduction in associated cost and ease of synthesis. Amino acids are rationally selected in line with a minimum pharmacophore required to permit antimicrobial activity [120]. Residues are selected to achieve an optimal lipophilic-charge balance. Typically, more active short peptides are the result of greater lipophilic bulk provided by non-proteinogenic groups. Strøm and co-workers investigated the effect of ultrashort antimicrobial peptides on a variety of nosocomial bacteria including methicillin resistant and sensitive Staphylococcus aureus, methicillin resistant Staphylococcus epidermidis and Escherichia coli and defined the minimum motif required for activity [106]. They hypothesized that activity in staphylococci required a combination of no more than two units of hydrophobic bulk and two units of charge. The Gram-negative rod required three units of bulk and at least two charged species for antibacterial activity to be observed. Such specificity allows ultrashort antimicrobial peptide therapy to be potentially tailored to causative microorganisms in infectious diseases. Harnessing this theory allowed the development of ultrashort lipopeptide variants. Peptides composed of four amino acids and a general sequence of KXXK (K = lysine, X = one of leucine, alanine, glycine, lysine or glutamic acid) were characterized for activity by the Shai group [108]. Aliphatic chains of various lengths were conjugated to these molecules. The resulting ultrashort lipopeptides were tested against a variety of bacteria, fungi, and yeast: Escherichia coli, Pseudomonas aeruginosa, Staphylococcus aureus, gentamicin-resistant Acinetobacter baumannii, Aspergillus fumigatus, Aspergillus flavus, and Candida albicans. This study resulted in three key findings. (1) The aliphatic side chain compensated for a reduced peptide chain length and contributed to hydrophobicity of the structure and resulting antimicrobial properties; (2) Substrate specificity is linked to the amino acid sequence or aliphatic chain length; (3) Many conventional antimicrobial peptides display activity against specific microbes. Some of the lipopeptides tested displayed activity against fungi as well as bacteria. Our own group used these finding together with the work of Bisht *et al*. to produce a series of ultrashort cationic lipopeptides [107]. Based on an ornithine-ornithine-tryptophan-tryptophan tetrapeptide amide motif, our group sequentially increased the lipophilic tail via conjugation of fatty acids to the ornithine terminus. C_12_-ornithine-ornithine-tryptophan-tryptophan displayed the most potent activity particularly against Gram-positive bacteria with complete eradication obtained within 24 h exposure against mature biofilms of Staphylococcus epidermidis (ATCC 35984) at concentrations as low as 15 µg/mL [45]. The group demonstrated it was possible to incorporate the peptide and the amphibian peptide maximin-4 into a poly(2-hydroxyethyl methacrylate) hydrogel in order to prevent short-term (24 h) Staphylococcus epidermidis biofilm adherence [46].

An alternative approach is to tag the end of the peptide residues with hydrophobic, bulky aromatics such as phenylalanine and tryptophan. Work on this area has involved ultrashort peptides composed of 4–7 amino acid residues. As with lipopeptides, these compounds are amphiphilic in nature and readily interact with target cell membranes. Of the tagged peptides investigated by Pasupuleti and colleagues [121], the most potent molecule, KNK10-WWWWW, displayed similar antimicrobial activity to human cathelicidin LL-37. Findings were similar for the phenylalanine-tagged peptides. Tagging also gave increased protection against proteolysis, providing an advantage for such compounds over the enzymatically degradable LL-37. This protection may be attributed to steric hindrance resulting from the bulky nature of the tags employed, preventing incorporation of the peptide into the protease active site.

Oligo-acyl-lysines (OAKs) are peptidomimetic molecules that mimic the primary structure and function of naturally occurring peptides. They are composed of acyl-lysines, with the acyl chain length determining hydrophobicity of the molecule and lysine conferring cationic charge for targeting of bacterial membranes or inhibition of cellular processes linked to intracellular DNA [122]. They can be linked to an inert resin to enhance the ability of OAKs to bind and capture bacteria for pathogen detection. A study conducted by Rotem and colleagues demonstrated that resin-bound OAKs have the potential to capture both Gram-positive and Gram-negative bacteria under continuous flow and stationary settings [123]. These molecules may be useful in terms of filtration of contaminated samples including drinking water in a hospital or community setting. Antimicrobial activity of OAKs has also been investigated *in vivo* highlighting their clinical potential. Livne demonstrated OAKs incorporating synergistic erythromycin had improved survival rates in neutropenic mice infected with multi-drug resistant *Escherichia coli* compared to erythromycin or OAK monotherapy [124]. Sarig determined the optimal lipid mixtures in the preparation of OAK-erythromycin cochleates, utilized for drug delivery [125]. Housing OAK molecules in a cochleate drug-delivery vehicle produced molecules with enhanced *in vivo* erythromycin efficacy. Cochleates are a drug delivery vehicle consisting of liposome-like molecules formed by a lipid-based supramolecular assembly of natural products (phosphatidylserine), negatively charged phospholipid and a divalent cation (calcium). The work of Sarig underlines the importance of the drug delivery vehicle in ensuring promising *in vitro* results for antimicrobial peptide-like molecules are translated efficiently to clinical practice. This is the greatest challenge in drug development and is particularly difficult with regard to peptides and proteins which suffer from poor solubility, pharmacokinetic properties, stability and antigenicity issues [126]. Hence antimicrobial peptides are often limited to topical use. The short peptides structures outlined possess many pharmaceutical advantages compared with larger peptide/protein molecules as their macromolecular structure allows for decreased recognition by proteases and antigens, with potential extension of their pharmacokinetic profile [127].

## 5. Self-Assembled Antimicrobial Peptides

The development of peptides that self-assemble upon exposure to environmental stimuli is of increasing interest in biomedicine. The ability to modify a peptide to assemble on cue provides the peptide with a range of desirable properties and potential applications including targeted drug delivery in the area of antimicrobials. For biomaterials, preventing the formation of a highly resistant biofilm phenotype is pivotal in preventing infection throughout the life of the material [128]. Harnessing environmental changes is a significant strategy to deliver activity when most required. Potential triggers for the formation of an inherently antimicrobial self-assembling hydrogel include: pH and ionic strength [50], temperature [129], light [130] and microbial enzymes [131].

### 5.1. Self-Assembly via Changes in pH and Ionic Strength

pH and ionic strength are the most commonly utilized methods to achieve assembly and disassembly of peptides on cue. Charge interactions are crucial to the assembly process and are influenced by the p*K*a of the peptide, the amino acid backbone and substituent functional groups. The effect of salt on pH-triggered assembly is also an important factor in determining the degree of ionic interactions [78]. The Schneider group is responsible for a large body of research in this area. They developed a synthetic peptide MAX-1, which adopted a beta(β)-hairpin structure due to the presence of a central V^D^PPT peptide motif (V = D-enantiomer of valine, ^D^P = D-enantiomer of proline, T = threonine) [132]. Lysine and valine amino acid residues flank the type-II β-turn structure in alternating sequences. Basic pH, above the p*Ka* of lysine’s primary amine R-group (pH > 9) resulted in self-assembly as intramolecular folding resulted in a hydrophobic valine core surrounded by a hydrophilic lysine surface. Lowering the pH, below the p*Ka* of lysine favored charge repulsion and disassembly. Addition of buffered saline (150 millimolar sodium chloride) or Dulbecco’s Modified Eagle’s Medium (165 millimolar sodium chloride) to 2% w/v of MAX-1 in water at pH 7.4 directed the formation of a biocompatible hydrogel. Formation of a hydrogel was due to screening of positive charged lysine moieties by chloride ions and alteration of the hydrophobic: charge balance allowing a decrease in solubility, therefore promoting assembly [133].

**Figure 3:**
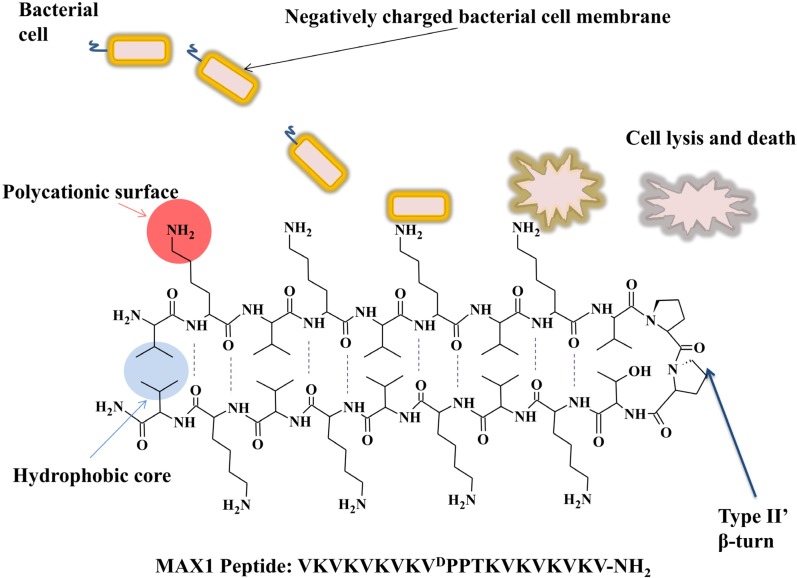
The antibacterial mechanism of action of self-assembling β-sheet cationic peptides using the example of MAX1 peptide developed by the Schneider group [132]. Basic pH, above the p*Ka* of lysine’s primary amine R-group (pH > 9), results in self-assembly of the primary peptide motif into a β-sheet secondary structure. The central V^D^PPT peptide forms a type II β-turn resulting in the formation of a hydrophobic valine core (blue) and a hydrophilic cationic lysine face (red). The primary amine (−NH_2_) R-groups of lysine protrude from the β-sheet structure forming a surface of polycationic character that is selective for negatively charged bacterial membranes. Adhesion and biofilm formation is prevented as bacterial membranes are compromised resulting in leakage of cell contents and bacterial cell death. In the hydrogel form the cationic groups may also displace divalent metal ions from the bacterial cell wall causing membrane disruption in biofilm cells, leading to cell death in both Gram-positive and –negative pathogens.

Replacing lysine with aspartic acid at amino acid position 15 (MAX-8) lowered the overall charge of the molecule by +2 and was sufficient to decrease gelation time from 30 min (for MAX-1) to 1 min [134]. Encapsulation of antimicrobial drugs and/or cells into this matrix could conceivably allow the targeted delivery of a hydrogel dressing for wound application via a syringe. This is especially true as MAX-1 was proven to display inherent antimicrobial properties against a broad spectrum of Gram-positive (*Staphylococcus epidermidis*, *Staphylococcus aureus* and *Streptococcus pyogenes*) and Gram-negative (*Klebsiella pneumoniae* and *Escherichia coli*) bacteria [132]. The results obtained highlight the role of the polycationic lysine surface in compromising negatively charged bacterial membranes resulting in bacterial cell death (Figure 3). The Schneider group altered the MAX1 template further to produce second generation cationic self-assembled antimicrobial hydrogels containing both cationic arginine and lysine residues [132]. MARG1, consisting of two arginine residues, was highly potent against methicillin resistant *Staphylococcus aureus*. A second peptide molecule, PEP6R, consisting of six arginines within its peptide primary structure, demonstrated broad spectrum activity against *Staphylococcus aureus*, *Escherichia coli* and multi-drug resistant *Pseudomonas aeruginosa*. Both were able to form hydrogels at physiological pH.

A related arginine rich peptide PEP8R (containing 8 arginine residues) was also created [135]. Variations of PEP8R were prepared, with arginine residues replaced by lysines to determine the optimal number of arginines required to achieve an antimicrobial effect. Decreasing the number of arginine residues formed PEP6R, a peptide that formed more rigid gels. The variants at all percentages tested were active against *Escherichia coli* and *Staphylococcus aureus*. Gels containing only two arginine residues demonstrated limited activity against Gram-negative *Escherichia coli*. Peptide derivatives were more active against Gram-positive bacteria. The presence of divalent ions such as calcium (Ca^2+^) reduced the peptides antimicrobial activity. The group concluded that in order to achieve optimal antimicrobial activity a minimum of four arginine residues were required.

Recent research by Liu and colleagues demonstrated the rational design of a pH dependent self-assembled antimicrobial peptide (ASCP1) consisting of two peripheral (KIGAKI)_3_-NH_2_ species conjugated to a central tetrapeptide linker (T^D^PPG) [50]. Similarly to the MAX peptides, the central motif allowed for the formation of a predominantly β-sheet structure with a central β-hairpin at pH greater than 10. The presence of twelve lysine primary amine side chains created sufficient electrostatic repulsion to prevent self-assembly. Elimination of these net charges via an increase in pH or addition of greater than 40 millimolar sodium chloride permits self-assembly. ASCP1 displayed inherent antibacterial properties against cultures of *Escherichia coli* after 36 h incubation. High bacterial loads (greater than 10^7^ colony forming units per mL) resulted in loss of inhibitory capacity. Again membrane disruption due to the presence of polycationic lysine residues was the most plausible mechanism for antibacterial activity.

RADA16 is one of the most comprehensively studied self-assembled peptides due to its ability to self-assemble at physiological pH [136]. Marketed commercially as PuraMatrix™ it has the ability to support cell growth and attachment leading to research into its use as a nanofiber scaffold to support wound healing. It has been hypothesized that functionalisation with antimicrobials and wound healing stimulants, such as epidermal growth factor, provide wound protection while encouraging wound closure [137]. Debnath and co-workers developed antimicrobial peptide amphiphiles which self-assemble at physiological pH [138]. These Fmoc pyridinium functionalised peptides were tested against a variety of Gram-positive (*Bacillus subtilis* and *Staphylococcus aureus*) and Gram-negative (*Escherichia coli* and *Pseudomonas aeruginosa*) bacteria, with minimum inhibitory concentrations determined. Compounds with the most broad-spectrum antimicrobial effect had hydrophobic phenylalanine closely attached to the Fmoc moiety. The close proximity of fluorenyl and phenyl moieties allowed an optimum hydrophobicity, described by the authors as the “threshold hydrophobicity”, to be obtained thus allowing significant activity against bacteria via membrane attack.

pH triggered self-assembly has potential to be utilized in antimicrobial therapies. Urinary catheter associated infections are synonymous with an increase in alkaline conditions due to the presence of the enzyme urease synthesized by Gram-negative *Proteus mirabilis*. Urease catalyses the breakdown of urea to ammonia via hydrolysis leading to increased pH at the urine-catheter interface and the precipitation of mineral salts such as calcium phosphate (hydroxyapatite) and magnesium ammonium phosphate (struvite). A combination of encrustation and biofilm formation leads to blockage of the catheter, with removal of the device necessary to resolve infection [139]. We hypothesize a role whereby an antimicrobial peptide may self-assemble at the device surface in response to an increase in pH (or the presence of urease) forming a protective barrier, reducing biofilm formation and encrustation thus preventing the need for catheter removal. This strategy would require the peptide molecule to be attached to the catheter surface, physicochemically stable at pH 7.4 and active against a broad spectrum of urinary pathogens, especially biofilm forms of *Proteus mirabilis*.

### 5.2. Photo-Activated Self-Assembly

Light may be used as an environmental trigger for the formation of hydrogels. These are typically formed when a water-soluble polymer and photo-initiator are exposed to a specific wavelength of light and crosslinking occurs. The resulting hydrogels display temporal and spatial resolution. Macromolecules are the preferred starting material and these are chosen to achieve the desired crosslinking and mechanical properties for the final hydrogel. The photo-initiator must be sufficiently reactive yet cytocompatible. Exposure to ultraviolet light triggers arrangement of a peptide into β-hairpins and subsequent self-assembly to a hydrogel occurs provided that the side chains permit this. Cysteine is hydrophobic in nature and easily functionalized. Therefore it is a useful side chain component for facilitating assembly. An example of this type of assembly is the 20-residue MAX7CNB peptide [130]. This caged (α- carboxy-2 nitrobenzyl) peptide is unfolded in ambient conditions but exposure to UV light triggers decaging and peptide folding to form a supramolecular hydrogel. There is no specific investigation in the literature for a peptide that induces self-assembly and antimicrobial activity in response to light. The use of light to stimulate antimicrobial delivery has been evaluated previously and therefore may serve as a valid mechanism for future research. McCoy and colleagues, developed an antimicrobial biomaterial containing tetracationic porphyrin, (tetrakis(4-*N*-methylpyridyl)porphyrin), which binds electrostatically with methacrylate groups of a methacrylic acid or a methyl methacrylate copolymer [140]. Visible light allowed the porphyrin to catalytically generate short-lived singlet oxygen at the device surface, with antimicrobial activity displayed against *Staphylococcus aureus* and *Pseudomonas aeruginosa*.

### 5.3. Thermo-Responsive Self-Assembly

Temperature can be used as a trigger to form higher assemblies (hydrogel structures) either through cooling or heating of molecules. The degree of hydrophobicity governs the temperature at which folding occurs. Typically, assembly of more hydrophobic molecules takes place at a lower temperature [141]. Schneider and colleagues modified their MAX-1 peptide, which forms a hydrogel at 25 °C and pH 9, by the replacement of two valine residues (at amino acid positions 7 and 16) with threonine [141]. This resulted in a peptide that formed hydrogel structures at higher temperatures (~60 °C). A replacement at position 16 only resulted in a gelation temperature of 40 °C, only slightly above that of normal body temperature, suggesting possible use as a thermo-responsive hydrogel in drug delivery. Liu’s ASCP1 peptide displayed similar self-supporting hydrogel structures at higher temperatures (~60 °C) [50]. Temperature change alters the solubility of the hydrophobic amino acid residues in these peptides, thus altering the hydrophobic: hydrophilic balance to favor decreased solubility. The process is thermally-reversible. Temperature-responsive gelation is not only limited to large peptide structures. Tang discovered replacing the terminal phenylalanine residue in Fmoc–FF with glycine (forming FmocFG) created a peptide that also gelled at increased temperatures [142]. Rheological analysis below its p*Ka* demonstrated self-assembly had occurred at 25 °C but only sufficient to produce a viscous solution. Gelation occurred between 55 and 80 °C which the authors attributed to dissolution of precipitate, formed due to a low pH, allowing homogenous hydrogels to be formed in solution. Further work by Tang studied glycine and leucine substituted Fmoc dipeptides [143]. In addition, employing pH and temperature to facilitate the formation of Fmoc hydrogels, FmocFG and FmocLG gelation proved also to be temperature-dependent. Both FmocFG and FmocLG hydrogels formed upon heating to 80 °C. Mechanical properties were retained upon subsequent cooling of heated peptides to 25 °C and 4 °C. FmocLG appeared to gel at 25 °C, with further heating and cooling of this gel to 4 °C forming stiffer hydrogel structures. This confirmed that a heating step was shown to improve the homogeneity of the samples without altering the topography of the self-assembled structures. Therefore, hydrophobicity of the peptide molecule, pH and homogeneity in aqueous solutions have to be taken into account in the design of a temperature triggered antimicrobial self-assembly peptide structure.

### 5.4. Bacterial Enzymatic Self-Assembly

Enzyme mediated self-assembly occurs as a result of catalysis or removal of a blocking group within the peptide primary sequence. It occurs under standard physiological conditions (pH and temperature) [144]. Enzymes such as proteases [145], phosphatases [49] and esterases [146], serve as viable molecules to drive peptide self-assembly. Catalysis occurs due to a thermodynamic shift resulting from condensation or hydrolysis of the peptide bond. In some cases both of these processes may occur in order to form peptide structures that are more stable. Research by Toledano demonstrated environmentally dependent protease triggered reverse hydrolysis of Fmoc amino acids [147]. Enzymes are selected, and the concentrations modified, in order to achieve optimum assembly under a defined set of conditions [148]. Enzymatic approaches to self-assembly are becoming more popular in research due to the abundance of bacterial enzymes, allowing tailored specificity for selected pathogens.

As outlined previously (Section 3), selection of specific amino acid residues and their corresponding properties is crucial in developing ultrashort peptides. Investigations into self-assembling ultrashort antimicrobial peptides are relatively novel but are increasing in the literature, especially in the area of enzyme triggered self-assembly. Removal or addition of one or two amino acid units has a larger influence on the overall properties of short peptide structures, including its solubility and therefore ability to self-assemble. One of the first examples of such research was conducted by the Xu group [49]. They were able to demonstrate that a strain of *Escherichia coli*, that overexpressed a phosphatase enzyme, was able to be selectively inhibited by a short naphthalene containing peptide. Dephosphorylation of soluble phosphorylated NapFFY(p), Nap^D^F^D^FY(p) and Nap-β^3^-HPhg-β^3^-HPhgY(p) (β^3^-HPhg: a β-amino acid named β^3^-homophenylglycine, p: phosphorylated) occurred in the cell cytoplasm causing inhibition of multiple intracellular processes resulting in a reduction in bacterial viability. Further work by the Xu group demonstrated how peptide self-assembly can be governed by a tyrosine-linked kinase/phosphatase switch [80]. Phosphorylation (via kinase) and dephosphorylation (via phosphatase) of the hydrogelator at the tyrosine terminus regulated the formation of supramolecular hydrogels. Gelation occurred *in vivo* in the presence of phosphatase enzyme, as removal of hydrophilic phosphate groupings increased the hydrophobicity of the molecule, driving self-assembly. The presence of phosphatases have been attributed to medical device related pathogens such as *Escherichia coli*, where alkaline phosphatase is present in the periplasmic space and therefore serves as a valid microbial target [149,150]. The Xu group [151] also produced a NapFF precursor conjugated to a β-lactam ring, capable of hydrogelation in response to the addition of β-lactamase enzymes from cell lysates of *Escherichia coli*. This mechanism has potential to be exploited for the detection of extended spectrum β-lactamases in a clinical setting and allowing screening of potential inhibitors.

Enzymatic assembly of ultrashort peptides, utilizing alkaline phosphatase, has been conducted more recently by the Ulijn group [131]. The Fmoc dipeptides (FY, YT, YS, YN and YQ in a FmocYpX-OH motif, where X = any amino acid) were central to the study, with tyrosine providing the hydroxyl grouping for phosphorylation. Phosphorous nuclear magnetic resonance (^31^P NMR) allowed determination of the rate of dephosphorylation and inhibition within defined areas of the bacterial cell and enabled the location of formed fibers to be identified. Treatment with alkaline phosphatase facilitates protonation of phosphate groups and the formation of higher assemblies. Peptide hydrogels formed over 24 h. Assembly was evident at a molecular level and there was evidence of β sheets, hydrogen bonding and π-stacking. Ability to assemble *in vivo* was investigated using media with and without the nucleoside inosine, which increases alkaline phosphatase synthesis two-fold. Bacterial cells were treated with a FmocFYp-OH precursor and HPLC analysis showed that the peptide moved into the hydrophobic environment of the bacterial cells. This demonstrated that assembly could occur *in vivo*. Treatment of *Escherichia coli* cells with other precursors showed similar results (self-assembly) however the effect of hydrophobicity on assembly and the resulting location of the formed peptides varied. More hydrophilic peptides were more likely to partition into the surrounding media rather than remaining within the hydrophobic environment of the bacterial cell. The findings indicated that formation of the peptides followed by movement out of the bacterial cells was sufficient to significantly reduce the number of viable bacterial cells. Therefore retention of the formed nanostructure within the cells was not essential to exert an antimicrobial effect. The intracellular and extracellular modes of action of these peptides reduce the ability of bacteria to develop resistance and bode well for their future development as therapeutics. The Ulijn group also employed the esterase subtilisin, obtained from the soil derived bacterium *Bacillus licheniformis*, as a mediator for assembly of Fmoc dipeptide methyl esters proving the versatility of the enzymatic approach to allow self-assembly [146].

## 6. Future Perspectivesand Translation of Peptide Self-Assembly to Antimicrobial Therapeutics

Challenges exist to the use of peptides as antimicrobial therapeutics. There is currently only one licensed self-assembled peptide therapeutic; the injectable β-sheet forming octapeptide lanreotide administered for the relief of neuroendocrine symptoms [152]. The licensing of lanreotide by the Food and Drug administration in 2007 provides great hope that similar molecules can be translated into therapeutics. In antimicrobial drug delivery there is increasing research into the utilization of self-assembled peptides as practical therapeutics. It is becoming increasingly relevant in the innovative production of antimicrobials and their delivery platforms. Peptide self-assembly has been utilized in combination with standardly employed antibiotics. Marchesan *et al.*, produced a macroscopic tripeptide hydrogel (^D^LFF) at physiological pH which incorporated the sparingly soluble antibiotic ciprofloxacin [153]. The hydrophobic tripeptide acted as a suitable drug delivery vehicle for the release of ciprofloxacin *in vitro*. Mild antibiofilm activity was demonstrated for the tripeptide alone against *Staphylococcus aureus*, *Escherichia coli*, and *Klebsiella pneumoniae*, with significant reduction in viability obtained via inclusion of 30% w/w ciprofloxacin.

Paladini and co-workers investigated the antimicrobial effects of silver incorporated into ultrashort Fmoc diphenylalanine (FmocFF) hydrogels on *Staphylococcus aureus*. [154]. Hydrogels (as detailed in Section 2) are commonly used in wound management and can be impregnated with antimicrobial agents, including silver. The nature of the hydrogels themselves creates a hydrated environment and protects the wound, creating an ideal environment for wound healing. Self-assembling peptide hydrogels such as those formed from assembly of FmocFF create a similar environment, making them ideal candidates for wound dressings. Paladini’s ‘silver-doped’ FmocFF hydrogels are composed of varying concentrations of silver nitrate (0.01%, 0.1%, 2% weight). The hydrogels produced were used to coat flax textiles and exposed to *Staphylococcus aureus* overnight. The work demonstrated the ability of FmocFF to disassemble and reassemble when added to the flax substrates. Scanning electron microscopy suggested that the higher the concentration of silver present, the more uniform the nature of the flax coverage. Precipitates formed with the 2% w/v gels thus homogeneity was not achieved. Antimicrobial studies demonstrated a reduction in bacterial numbers with an increased silver concentration. Bacterial adhesion and biofilm formation was also investigated. Incorporation of silver was necessary to prevent biofilm formation and the minimum concentration of silver needed to achieve this effect was 0.1% weight.

The amphipathic peptide, FmocFFECG, contains both hydrophobic and hydrophilic moieties. The presence of carboxylic acid functionalities allowed adsorption of silver nanoparticles into the peptide motif [155]. Activity was proven against Gram-positive *Bacillus subtilis* and Gram-negative *Escherichia coli* for up to 30 days. Stability was also increased compared with silver nanoparticles alone suggesting the peptide hydrogel protects silver nanoparticles from oxidation. The hydrophobic character of ultrashort self-assembling peptides may also allow the incorporation of other highly lipophilic antimicrobial drugs.

Peptides also provide access to infections in the brain. Research by Liu demonstrated how the peptide CG_3_R_6_TAT (C = hydrophobic cholesterol, G = glycine spacers, R = cationic arginine residues, TAT = minimal amino acid sequence required for membrane translocation) assembles to form micelles with a hydrophobic cholesterol core and hydrophilic peptide exterior [156]. This peptide displays antimicrobial activity against a range of Gram-positive and Gram–negative bacteria. Ability to cross the blood brain barrier and exert an antimicrobial effect was also demonstrated *in vivo* in an infected *Staphylococcus aureus* induced meningitis rabbit model. This work highlights the ability of assembled peptides as tailored molecules to achieve targeted antimicrobial effects in challenging areas of drug delivery.

Key problems associated with peptide design namely, high manufacturing costs and susceptibility to enzymatic degradation, mean there is still a need to develop compounds that have similar properties to conventional antimicrobial peptides in physiological conditions namely: cationic charge, hydrophobicity and amphiphilic nature to enable antimicrobial action and self-assembly. Peptidomimetics and other synthetic oligomers are thus coming to the forefront of current and future research plans. The design of ultrashort peptides has the potential to create functional peptide molecules with a relatively reasonable manufacturing cost. Our own group has moved research forward in this area with the synthesis of a variety of ultrashort and more cost-effective cationic naphthalene containing peptide hydrogels, which are highly active against biofilm forms of biomaterial related pathogens [157]. The very nature of peptides and their proven activity against pathogenic biofilms renders them useful compounds for prevention and management of nosocomial infections and wound healing. Further development of peptidomimetics will provide novel biomaterial platforms and the development of new molecules with tunable chemical and mechanical properties.
